# Infrared identification of the Criegee intermediates *syn*- and *anti*-CH_3_CHOO, and their distinct conformation-dependent reactivity

**DOI:** 10.1038/ncomms8012

**Published:** 2015-05-11

**Authors:** Hui-Yu Lin, Yu-Hsuan Huang, Xiaohong Wang, Joel M. Bowman, Yoshifumi Nishimura, Henryk A. Witek, Yuan-Pern Lee

**Affiliations:** 1 Department of Applied Chemistry and Institute of Molecular Science, National Chiao Tung University, 1001, Ta-Hsueh Road, Hsinchu 30010, Taiwan; 2 Department of Chemistry and Cherry L. Emerson Center for Scientific Computation, Emory University, Atlanta, Georgia 30322, USA; 3 Institute of Atomic and Molecular Sciences, Academia Sinica, Taipei 10617, Taiwan

## Abstract

The Criegee intermediates are carbonyl oxides that play critical roles in ozonolysis of alkenes in the atmosphere. So far, the mid-infrared spectrum of only the simplest Criegee intermediate CH_2_OO has been reported. Methyl substitution of CH_2_OO produces two conformers of CH_3_CHOO and consequently complicates the infrared spectrum. Here we report the transient infrared spectrum of *syn*- and *anti*-CH_3_CHOO, produced from CH_3_CHI + O_2_ in a flow reactor, using a step-scan Fourier-transform spectrometer. Guided and supported by high-level full-dimensional quantum calculations, rotational contours of the four observed bands are simulated successfully and provide definitive identification of both conformers. Furthermore, *anti*-CH_3_CHOO shows a reactivity greater than *syn*-CH_3_CHOO towards NO/NO_2_; at the later period of reaction, the spectrum can be simulated with only *syn*-CH_3_CHOO. Without NO/NO_2_, *anti*-CH_3_CHOO also decays much faster than *syn*-CH_3_CHOO. The direct infrared detection of *syn*- and *anti*-CH_3_CHOO should prove useful for field measurements and laboratory investigations of the Criegee mechanism.

## Abstract (web summary)

Criegee intermediates—formed by the breakdown of alkanes in the atmosphere—are difficult to identify in the gas phase. Here, the authors report the infrared spectrum of the Criegee intermediate CH_3_CHOO and identify both the *syn*- and *anti*-conformers.

## Graphical abstract

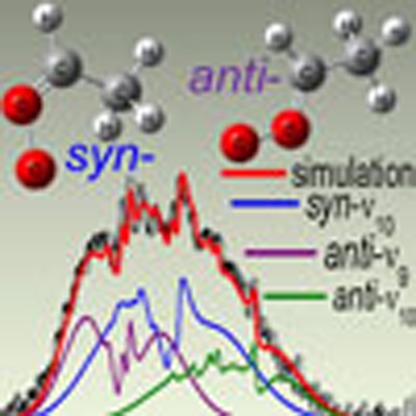

Recent advances in the direct detection of the Criegee intermediates have stimulated active research on their structures, spectroscopy, reactions and implications for atmospheric chemistry^1^. The Criegee intermediates are carbonyl oxides that are produced during the reactions of ozone (O_3_) with unsaturated hydrocarbons; they play a central role in controlling the atmospheric budget of OH, organic acids and secondary organic aerosols, especially under low-light conditions.

These Criegee intermediates have eluded detection in the gaseous phase until recently because of their great reactivity and small concentration. Previous understanding of the reactivity of Criegee intermediates, including reaction mechanisms and rate coefficients, was hence subject to large uncertainties, because only indirect experimental evidence was available. Welz *et al*.^3^ produced the simplest Criegee intermediate, formaldehyde oxide (CH_2_OO), in a flow cell and confirmed its identification with tunable vacuum-ultraviolet photoionization mass spectrometry^2^. Following the reaction scheme of CH_2_I+O_2_→I+CH_2_OO initially developed by Welz *et al*.^3^, CH_2_OO has now been detected with ultraviolet depletion^4^, ultraviolet absorption^5^,^6^, infrared absorption^7^ and microwave spectroscopy^8^,^9^. The kinetics of reactions of CH_2_OO with various atmospheric species have been directly investigated with some of these detection methods^3^,^10^,^11^,^12^,^13^,^14^,^15^.

In the atmosphere, O_3_ reacts with larger alkenes and produces more complex Criegee intermediates RR′COO; the nature and location of the substituents R/R′ are predicted to affect their reactivity^16^. For example, the OH yield increases from ∼10% for O_3_+C_2_H_4_ to ∼60% for O_3_ + *trans*-2-butene^17^,^18^. The methyl-substituted Criegee intermediate, acetaldehyde oxide (CH_3_CHOO), is an intermediate of reactions O_3_ + 2-alkenes (such as *trans*-2-butene) and serves as a prototype to understand various fundamental issues in larger Criegee intermediates. CH_3_CHOO exists in two conformers, *syn*-CH_3_CHOO and *anti*-CH_3_CHOO (Supplementary Fig. 1), with the former more stable than the latter by ∼15 kJ mol^−1^ (refs 19, 20). Because of a large barrier ∼160 kJ mol^−1^, the interconversion between *syn*-CH_3_CHOO and *anti*-CH_3_CHOO is unlikely. Taatjes *et al*.^21^ employed the reaction CH_3_CHI + O_2_ to produce CH_3_CHOO and detected both conformers with photoionization. By employing photoionization at energies 9.37 and 10.5 eV to detect predominantly *anti*-CH_3_CHOO and *syn*-CH_3_CHOO, respectively, these authors reported that *anti*-CH_3_CHOO is significantly more reactive towards H_2_O and SO_2_ than is *syn*-CH_3_CHOO.

Although photoionization can distinguish these two conformers of CH_3_CHOO via distinct ionization energies, a small contribution from the other conformer is unavoidable. The ultraviolet depletion^22^ and ultraviolet absorption experiments^20^ yielded a broad feature without structure and provided no information about the conformation of the CH_3_CHOO carrier. Recently Sheps *et al*.^23^ separated the ultraviolet spectra of two possible conformers based on their distinct reactivity towards H_2_O and SO_2_, but the two spectra were broad and overlapping with each other. Although microwave spectroscopy was successful in providing structural information^24^, to employ it for monitoring these species in laboratory kinetic experiments is difficult. Liu *et al*.^25^ employed infrared activation of cold CH_3_CHOO to produce OH, which was detected with laser-induced fluorescence, and assigned several absorption features in the region 5,600–6,100 cm^−1^ to be the CH-overtone and combination bands of *syn*-CH_3_CHOO; no bands of *anti*-CH_3_CHOO were identified. It is thus desirable to develop an alternative spectral method that can be applied conveniently and can distinguish clearly these two conformers of CH_3_CHOO.

We have demonstrated that coupling a step-scan Fourier-transform infrared (FTIR) spectrometer with a multipass absorption cell enables the recording of temporally resolved infrared absorption spectra of the simplest Criegee intermediate, CH_2_OO (ref. 7); five distinct absorption bands of CH_2_OO were clearly identified to provide a simple and direct detection method.

Here we report a further application of this technique to characterize the infrared absorption of methyl-substituted Criegee intermediate CH_3_CHOO in two conformations and to demonstrate the conformation-dependent reactivity towards its self-reaction and reaction with NO/NO_2_.

## Results

### Spectral analysis of CH_3_CHOO

Even though only one H atom of CH_2_OO was replaced with a methyl group to form CH_3_CHOO, the infrared spectrum of the latter is expected to be much more complicated, because both *syn*- and *anti*-conformers contribute to the infrared absorption. In addition, the methyl group introduces low-energy vibrational modes such as the CH_3_ torsion (internal rotation), which are populated with several vibrational quanta even at ambient temperatures; hot bands might consequently play important roles in the observed spectrum, as it was demonstrated in the spectrum of CH_2_BrOO (ref. 26). Furthermore, the internal rotation of the methyl moiety may introduce torsional splitting in vibrational bands. Consequently, spectral identification and simulation of observed bands of CH_3_CHOO are expected to be difficult. In this respect, sophisticated quantum-chemical calculations are essential to assist the spectral simulation and assignments.

**Figure 1:**
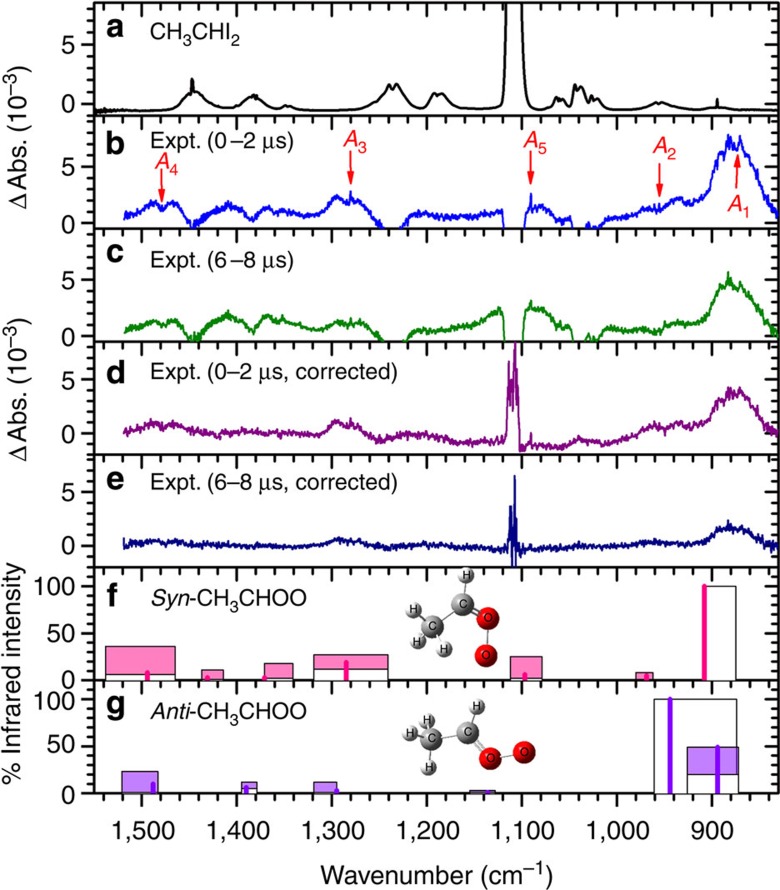
Comparison of observed spectra with predicted stick spectra. (**a**) Absorption spectrum of a flowing mixture of CH_3_CHI_2_/N_2_/O_2_ (1/13/288, 84 Torr) before photolysis. (**b**) Difference spectra recorded 0–2 μs and (**c**) 6–8 μs after irradiation of the sample at 308 nm. (**d**) Corrected spectra recorded 0–2 μs and (**e**) 6–8 μs after subtraction of the spectrum recorded at 16.0–19.8 μs and removal of the contributions of the precursor CH_3_CHI_2_ and stable product acetaldehyde. Resolution of all spectra is 0.5 cm^−1^. New features are marked with arrows and labelled as *A*_1_–*A*_5_. (**f**) Possible ranges of anharmonic vibrational wavenumbers and infrared intensities of *syn*-CH_3_CHOO and (**g**) *anti*-CH_3_CHOO predicted with various methods (Supplementary Tables 1 and 2) shown as filled boxes; those predicted with the MULTIMODE method are shown with thick lines.

We employed a step-scan FTIR spectrometer coupled with a multi-reflection White cell to record time-resolved infrared spectra. The laser beam at 308 nm photodissociated a flowing mixture of 1,1-diiodoethane (CH_3_CHI_2_) and O_2_ to produce CH_3_CHI, which subsequently reacted with O_2_ to form CH_3_CHOO. The partial infrared absorption spectrum (830–1,550 cm^−1^) of a flowing mixture of CH_3_CHI_2_/N_2_/O_2_ (1/13/288, 84 Torr) at 328 K exhibits an intense absorption line of CH_3_CHI_2_ near 1,108 cm^−1^ and some much weaker ones near 894, 956, 1,024, 1,041, 1,061, 1,190, 1,236, 1,383 and 1,447 cm^−1^ (Fig. 1a). On irradiation with light at 308 nm, the absorption of CH_3_CHI_2_ decreased because of photolysis, whereas new bands marked *A*_1_–*A*_5_ appeared in the difference spectrum recorded 0–2 μs after laser irradiation (Fig. 1b). The *A*_5_ band was partially interfered with by the absorption of the precursor and the product acetaldehyde, but its sharp *Q*-branch is quite characteristic and can be readily recognized. These new lines decreased in intensity, as shown in Fig. 1c where 6–8 μs was recorded after ultraviolet irradiation and disappeared after ∼25 μs. To minimize interference from other products, we subtracted the spectrum recorded during 16.0–19.8 μs from these two spectra and stripped the contributions from the precursor and acetaldehyde, as shown in Fig. 1d,e.

As photolysis of CH_3_CHI_2_ produces mainly CH_3_CHI (ref. 27) and because this radical reacts readily with excessive O_2_ in the system^28^, possible carriers of the observed new bands include conformers of CH_3_CHOO and vinyl hydroperoxide (C_2_H_3_OOH), methyldioxirane and the stabilized adduct CH_3_CHIOO. The structures of these species are shown in Supplementary Fig. 1. For all species except CH_3_CHIOO, MP2, B3LYP and NEVPT2(8,8) methods were employed to predict their vibrational wavenumbers and infrared intensities. For CH_3_CHIOO, the NEVPT2(1,1) method was used. Furthermore, the most sophisticated predictions were performed for *syn*- and *anti*-CH_3_CHOO with high-level, full-dimensional quantum calculations, using the MULTIMODE method.^29^,^30^,^31^ The vibrational wavenumbers and infrared intensities of *syn*- and *anti*-CH_3_CHOO calculated with these theoretical methods are given in Supplementary Tables 1 and 2. Those of methyl dioxirane, *trans*- and *cis*-vinyl hydroperoxide and *syn*- and *anti*-CH_3_CHIOO are listed in Supplementary Tables 3–5, respectively. There is good consistency among these calculations, but there are significant variations in the calculated anharmonic vibrational wavenumbers and infrared intensities among these calculations. To indicate this, we show the ranges of values as filled rectangles, while the values predicted with the MULTIMODE method are depicted with thick lines in Fig. 1f,g, respectively, for *syn*- and *anti*-CH_3_CHOO. Possible ranges of vibrational anharmonic wavenumbers and intensities of CH_3_CHIOO, methyl dioxirane and vinyl hydroperoxide predicted with various methods are compared with the observed spectrum in Supplementary Fig. 2. A comparison of the observed new spectral features with those predicted theoretically excludes the possibility of assigning the observed spectral features *A*_1_–*A*_5_ to the carriers other than CH_3_CHOO.

As shown in Fig. 1f and Supplementary Table 1, the more stable *syn*-CH_3_CHOO conformer is predicted from the MULTIMODE calculations to have more intense absorptions near 908 (100), 969 (5), 1,097 (6), 1,285 (19) and 1,494 (8) cm^−1^; the numbers in parentheses give relative infrared intensity of each vibration. The observed new features near 871 (100), 956, 1,091 (10), 1,281 (40) and 1,477 (30) cm^−1^ agree satisfactorily with these predicted values. The predicted pattern of intense lines of *anti*-CH_3_CHOO near 894 (49), 944 (100), 1,295 (3) and 1,488 (10) cm^−1^ agrees less satisfactorily with the observed spectrum. We show below that the contribution from the *anti*-CH_3_CHOO conformer, although less significant, is important for explaining the overall shape of the spectrum and is of primary importance for explaining some of the fine-structure spectral features of the spectrum.

### Simulation of rotational contours

**Figure 2:**
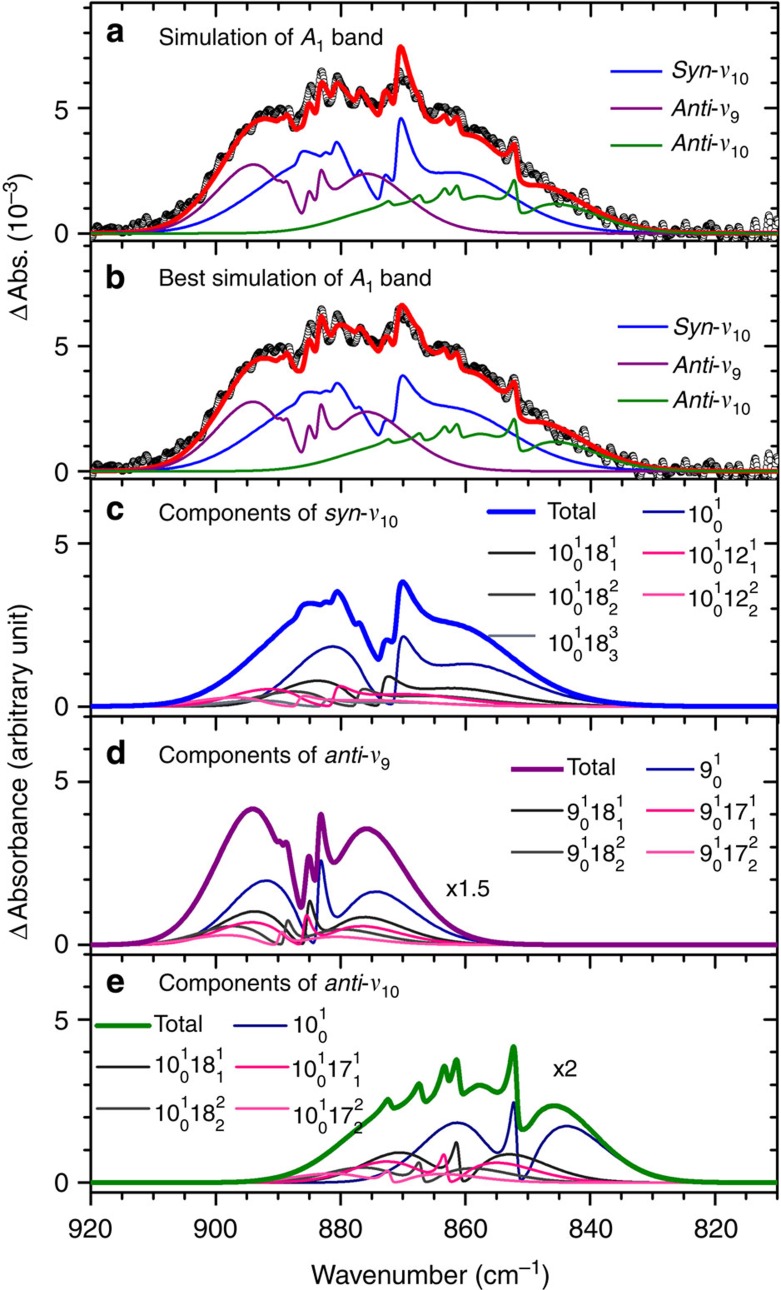
Spectral simulation of band *A*_1_. (**a**) Comparison of experimental data (open circles, recorded 0–2 μs) with spectrum simulated according to theoretical predictions (thick red solid line) and (**b**) the best simulated spectrum (thick red solid line) with slightly modified parameters; contributions of *ν*_10_ of *syn*-CH_3_CHOO, and *ν*_9_ and *ν*_10_ of *anti*-CH_3_CHOO are shown with thin lines. Resolution is 0.5 cm^−1^. (**c**) Contributions of fundamental and hot bands of *ν*_10_ of *syn*-CH_3_CHOO, (**d**) *ν*_9_ of *anti*-CH_3_CHOO and (**e**) *ν*_10_ of *anti*-CH_3_CHOO. Detailed positions and relative intensities are listed in Supplementary Table 6. Spectral width of simulation is 0.64 cm^−1^. The transitions of hot bands are expressed with 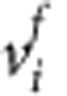 in which *v* is the vibrational mode number, and *i* and *f* are vibrational quantum numbers of the lower and upper states, respectively.

Further support for the presented assignment comes from analysis of the rotational contours of the observed bands. The rotational contour of the *A*_1_ band near 871 cm^−1^ recorded in the interval of 0–2 μs has several characteristic peaks; it cannot be simulated with a single band and was deconvoluted with guidance from calculations. The experimental data are presented with open circles and the resultant spectrum, simulated according to ratios of rotational constants of the excited and ground states predicted with the MULTIMODE method and the experimental rotational constants of the ground state, is shown as a thick solid line in Fig. 2a; the agreement is satisfactory. Figure 2b presents a comparison of experimental data with the spectrum simulated according to slightly modified ratios of rotational constants for an improved fit. For details of simulation using the PGopher programme^32^, see Supplementary Methods. This simulated feature consists of three bands: a dominant OO-stretching (*ν*_10_) band of *syn*-CH_3_CHOO at 871.2 cm^−1^ and two smaller bands of OO-stretching (*ν*_9_) and OO-stretching mixed with CH_2_ wagging (*ν*_10_) modes of *anti*-CH_3_CHOO at 883.7 and 851.8 cm^−1^, respectively, shown as thin lines in Fig. 2a,b; the fundamental *Q*-branches of these three modes correspond well with observed distinct peaks; hence, their vibrational wavenumbers can be determined accurately. It is found that several hot bands associated with the *ν*_18_ (208 cm^−1^) and *ν*_12_ (314 cm^−1^) low-energy vibrational modes of *syn*-CH_3_CHOO and with the *ν*_18_ (156 cm^−1^) and *ν*_17_ (255 cm^−1^) low-energy vibrational modes of *anti*-CH_3_CHOO contribute significantly to the observed spectral features. The individual contributions of fundamental and hot bands for these three vibrational modes are shown in Fig. 2c–e; the transitions of hot bands are expressed as 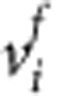 in which *v* is the vibrational mode number, and *i* and *f* are vibrational quantum numbers of the lower and upper states, respectively. Detailed positions and relative intensities are listed in Supplementary Table 6. This simulation implies that (1) the hot bands are all blue shifted from the fundamental; the unusual but necessary blue shifts of about 2 and 10 cm^−1^ for the hot bands involving *ν*_18_ and *ν*_12_ of *syn*-CH_3_CHOO, respectively, agree qualitatively with the theoretical predictions (Supplementary Table 6). The calculations also indicate that torsional splitting is small for all vibrational modes so that it has no consequence on our spectral simulation. (2) At 328 K, assuming a Boltzmann distribution and that the infrared intensities of hot bands are the same as that of the fundamental band (in qualitative accord with calculations), observed relative intensities of these hot bands imply energies of the first excited states of *ν*_18_ and *ν*_12_ modes of *syn*-CH_3_CHOO and those of *ν*_18_ and *ν*_17_ of *anti*-CH_3_CHOO to be ∼193, 282, 149 and 239 cm^−1^, respectively, which is consistent with theoretical predictions (Table 1 and Supplementary Tables 1 and 2). (3) The population fraction of *anti*-CH_3_CHOO is 0.30 and 0.38 at 328 K, respectively, if infrared intensities predicted with the B3LYP and MULTIMODE methods are used. This fraction is consistent with a value of ∼0.30 derived from ultraviolet experiments^23^, but greater than values ∼0.20 from microwave experiments^24^ and ∼0.10 from photoionization experiments^21^.

**Figure 3:**
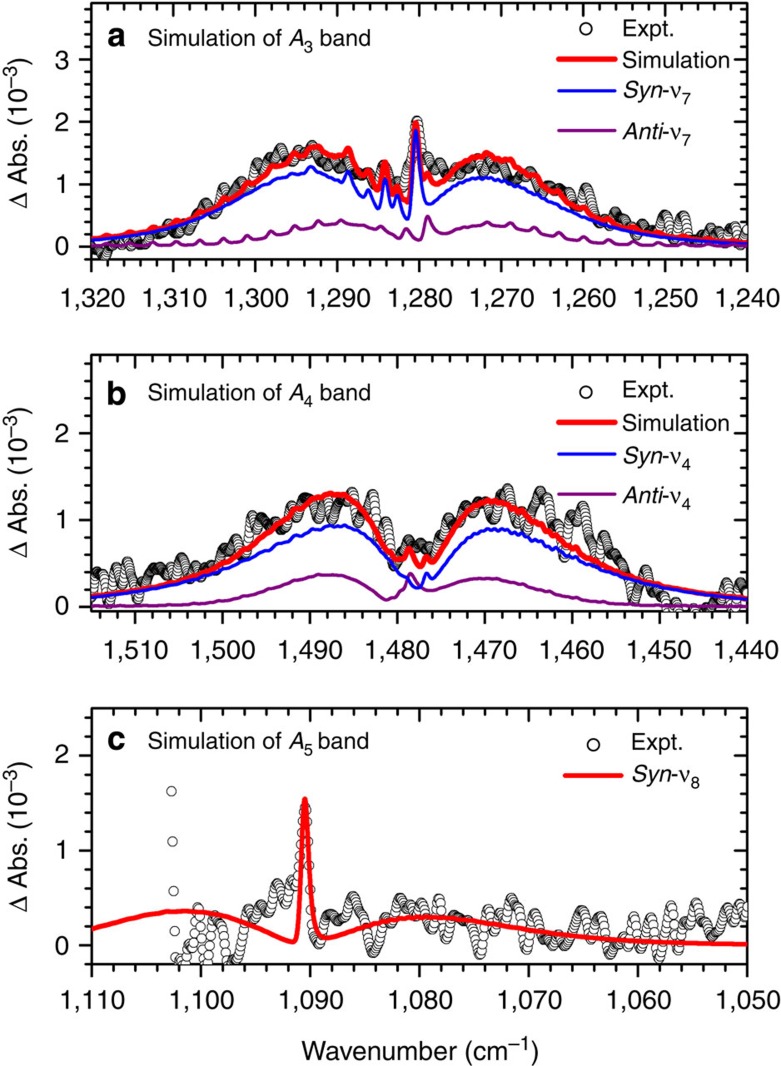
Spectral simulation of bands *A*_3_ to *A*_5_. (**a**) Comparison of experimental data (open circles, recorded 0–4 μs) with simulated spectrum (thick red solid line) for band *A*_3_; contributions of *ν*_7_ bands of *syn*-CH_3_CHOO and *anti*-CH_3_CHOO are shown with thin lines. (**b**) Comparison for band *A*_4_; contributions of *ν*_4_ bands of *syn*-CH_3_CHOO and *anti*-CH_3_CHOO are shown with thin lines. (**c**) Comparison of band *A*_5_ (recorded 0–2 μs); only the *ν*_8_ band of *syn*-CH_3_CHOO contributes. Spectral width of simulation is 0.64 cm^−1^.

The weaker bands *A*_3_ and *A*_4_ can be simulated likewise with contributions of *syn*- and *anti*-CH_3_CHOO, as presented in Fig. 3. Band *A*_3_ has a prominent *Q*-branch at 1,280.8 cm^−1^, which is assigned to the HCO bending coupled with the CO-stretching (*ν*_7_) mode of *syn*-CH_3_CHOO. A second feature with a weaker *Q*-branch at 1,279.4 cm^−1^ is assigned to the corresponding *ν*_7_ mode of *anti*-CH_3_CHOO (Fig. 3a). Band *A*_4_ has prominent *P*- and *R*-branches; this band is assigned to the CO-stretching mode coupled with the HCO bending (*ν*_4_) mode of *syn*-CH_3_CHOO. The observed weak *Q*-branch arises from a small contribution of the *ν*_4_ mode of *anti*-CH_3_CHOO. The contributions of fundamental and hot bands to these features are presented in Supplementary Figs 3 and 4. The *a*- and *b*-type spectra of related vibrational modes of *syn*- and *anti*-CH_3_CHOO are presented in Supplementary Figs 5 and 6, respectively. As band *A*_2_ is rather weak and subject to interference from absorption of C_2_H_4_, we could only estimate its position to be ∼956 cm^−1^ from the *Q*-branch and assign it to the *ν*_9_ mode of *syn*-CH_3_CHOO. Band *A*_5_ suffers from partial interference due to the precursor, but the prominent *Q*-branch indicates that this band is due to *syn*-CH_3_CHOO and not *anti*-CH_3_CHOO. The observed position at 1,090.6 cm^−1^ is much closer to the predicted anharmonic vibrational wavenumber of 1,097 cm^−1^ for *syn*-CH_3_CHOO than the value 1,136 cm^−1^ for *anti*-CH_3_CHOO.

As shown in Table 1, the agreement between observed vibrational wavenumbers of *syn*- and *anti*-CH_3_CHOO, and those calculated with the MULTIMODE method is quite satisfactory with differences <17 cm^−1^, except for the OO-stretching mode, which has differences of 37 cm^−1^ for *syn*-CH_3_CHOO and 60 cm^−1^ for *anti*-CH_3_CHOO. A similar shift between experiment and MULTIMODE calculations was reported previously for CH_2_OO for an analogous band^33^. Conceivably, the origin of the deviation is, as in CH_2_OO, a slight deficiency in the level of electronic structure theory which is more sensitive to the OO-stretching mode.

Our observations also conform to an expectation that similar to CH_2_OO, CH_3_CHOO has a significant zwitterionic character with a strengthened C−O bond and a weakened O−O bond. The observed wavenumber of the OO-stretching mode of *syn*-CH_3_CHOO near 871 cm^−1^ is smaller than the corresponding value 908 cm^−1^ of CH_2_OO, consistent with theoretical predictions showing that the length of the O–O bond increases from 1.349 Å in CH_2_OO to 1.380 Å in *syn*-CH_3_CHOO.

### Conformation-dependent reactivity

**Figure 4:**
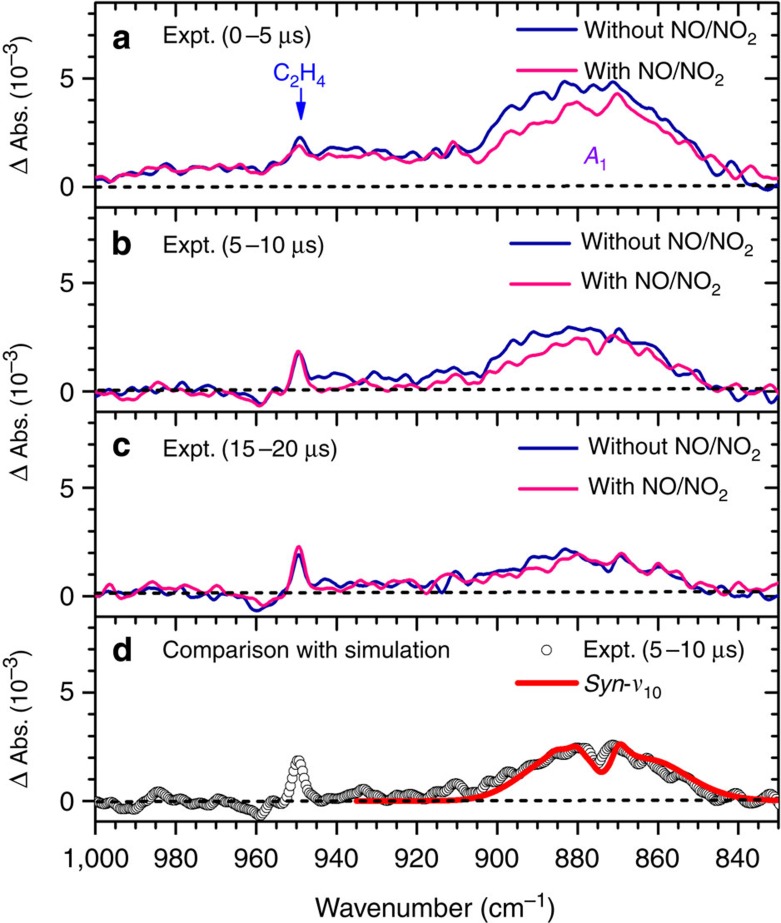
Comparison of spectra recorded with and without added NO/NO_2_. (**a**) A flowing mixture of CH_3_CHI_2_/N_2_/O_2_ (1/21/129, 90 Torr) with and without added NO/NO_2_ (∼3.0/0.18 Torr) recorded 0–5 μs, (**b**) 5–10 μs and (**c**) 15–20 μs after irradiation at 248 nm. (**d**) Comparison of experimental data (open circles) with simulated spectrum (thick red solid line) of *ν*_10_ of *syn*-CH_3_CHOO. Spectral width of simulation is 2.6 cm^−1^.

Further support for this assignment of the *A*_1_ band comes from a comparison of low-resolution spectra recorded in experiments with and without NO/NO_2_. Taatjes *et al*.^21^ reported that *anti*-CH_3_CHOO is more reactive towards H_2_O, SO_2_ and NO_2_ than *syn*-CH_3_CHOO, with the effect much more significant for the former two. A direct test with H_2_O in our system is difficult because of the intense infrared absorption of H_2_O and the hygroscopic KBr windows we employed. A test with SO_2_ is also infeasible, because the windows were contaminated by the products easily. Instead, we added NO/NO_2_ to explore the differences. As shown in Fig. 4a, the difference between bands *A*_1_ in the range 870–910 cm^−1^ recorded in experiments with and without adding NO/NO_2_ is significant during the interval 0–5 μs and the difference in the range 830–870 cm^−1^ is smaller but non-negligible. The differences in these two regions are consistent with the contributions of *ν*_9_ and *ν*_10_ bands of *anti*-CH_3_CHOO shown in Fig. 2a. This difference in spectra recorded in experiments with and without NO/NO_2_ diminished at a later period and the spectrum recorded 5–10 μs after photolysis in the experiment with NO/NO_2_ (Fig. 4b) agrees with that simulated for *syn*-CH_3_CHOO only, as shown in Fig. 4d; this provides additional support that our simulation of these two conformers is reliable and most *anti*-CH_3_CHOO was consumed within 10 μs.

Further indication of the greater reactivity of *anti*-CH_3_CHOO than that of *syn*-CH_3_CHOO comes from the observation of a more rapid decrease of peaks at 851.8 and 883.7 cm^−1^ (assigned to *anti*-CH_3_CHOO) relative to that at 871.2 cm^−1^ (assigned to *syn*-CH_3_CHOO) in the earlier stage of reaction. Without NO/NO_2_, nearly all *anti*-CH_3_CHOO diminished and only *syn*-CH_3_CHOO survived after 10 μs; *syn*-CH_3_CHOO became nearly diminished after 25 μs. The decay of *syn*-CH_3_CHOO is likely to be mainly due to its self-reaction, similar to that observed for CH_2_OO (refs 7, 14, 15). In Supplementary Fig. 7, we plotted the reciprocal absorbance (*I*^−1^) integrated from 850 to 900 cm^−1^ for band *A*_1_ as a function of reaction period; the integrated absorbance *I* is proportional to concentration. The slope at the initial stage of reaction is twice that at a later stage of reaction. A qualitative analysis indicates that the overall removal of *anti*-CH_3_CHOO could be up to 30 times faster than that of *syn*-CH_3_CHOO; this probably reflects the difference in reactivity of these two conformers associated with some reactions with themselves or with other species. We are unable to derive absolute concentration of CH_3_CHOO, because the infrared probe volume does not follow the photolysis volume in our experiments^14^; hence, accurate rate coefficient cannot be determined. In any case, it is clear that in the CH_3_CHI + O_2_ system the reactivity of *anti*-CH_3_CHOO is much greater than that of *syn*-CH_3_CHOO, so that most *anti*-CH_3_CHOO was consumed within 10 μs under our experimental conditions. Future theoretical investigations on this distinct conformation-dependent reactivity will provide some insight on this important observation.

## Discussion

We recorded the transient infrared spectrum of both *syn*- and *anti*-CH_3_CHOO, produced from CH_3_CHI + O_2_ in a flow reactor, using a step-scan Fourier-transform spectrometer. The rotational contours, vibrational wavenumbers and relative infrared intensities of these observed bands provide definitive identification of both conformers; the rotational contours are critical in distinguishing these conformers. The assignments and spectral simulation are guided and supported by high-level full-dimensional quantum calculations. We found that a*nti*-CH_3_CHOO is more reactive towards NO/NO_2_ than *syn*-CH_3_CHOO. Without NO/NO_2_, a*nti*-CH_3_CHOO also decays much more rapidly than *syn*-CH_3_CHOO. This feature shall enable one to prepare nearly pure *syn*-CH_3_CHOO either by adding NO/NO_2_ or by waiting till a later reaction period. This distinct conformation-dependent reactivity is of fundamental interest and is also important in modelling reactions involving Criegee intermediates both in laboratory and in the atmosphere.

Our infrared identification provides an alternative way to probe CH_3_CHOO directly in the atmosphere or in laboratory experiments. Although nearly all observed bands of *anti*-CH_3_CHOO overlapped with *syn*-CH_3_CHOO, the *Q*-branch of *ν*_8_ near 1,090.6 cm^−1^ is contributed solely by *syn*-CH_3_CHOO, and that of *ν*_7_ near 1,280.8 cm^−1^ is also dominated by *syn*-CH_3_CHOO; they can be unambiguously detected with a narrow-width infrared laser. This convenient detection scheme will be useful in providing information on the exact mechanism and detailed kinetics of reactions involving CH_3_CHOO.

## Methods

### Experimental

The White cell of base path length 15 cm and effective absorbing path length 3.6 m was coupled with a step-scan FTIR spectrometer (Bruker, Vertex 80v) and served as a flow reactor. The flow reactor has volume ∼1,370 cm^3^ and accommodates two rectangular quartz windows (3 × 12 cm^2^) on opposite sides of the cell, to allow the photolysis beam to propagate through the cell. The photolysis light at 308 nm was generated from a XeCl excimer laser (Coherent, CompexPro 102F, 11 Hz, ∼170 mJ per pulse, beam size 2.9 × 1.2 cm^2^). In earlier experiments, light at 248 nm from a KrF excimer laser (Coherent, CompexPro 102F, 11 Hz, ∼200 mJ per pulse, beam size 1.5 × 1.0 cm^2^) was used. The photolysis beam passed the White cell and was reflected five times with two external mirrors, to enhance the photolysis. With appropriate optical filters to allow passage of light in a narrow spectral region, we performed undersampling to decrease the size of the interferogram, hence the duration of data acquisition. The infrared probing light was detected with a HgCdTe detector from which dc- and ac-coupled signals were recorded at each scan step on irradiation with the photolysis laser. These signals were sent either directly to the external 14-bit digitizer with resolution of 4 or 10 ns. For the ac signal, 5,000 data points at 4 (10) ns intervals were acquired to cover a period of 19.8 (48.0) μs after photolysis. These signals were typically averaged over 15 laser shots at each scan step. For spectra in the range 753–1,504 cm^−1^ at resolution 0.5 cm^−1^, 3,046 scan steps were completed in ∼1.5 h. For spectra in the range 1,089−1,633 cm^−1^ at resolution 0.5 cm^−1^, 2,206 scan steps were completed in ∼1 h. The spectral width (full width half maximum) at this resolution after apodization is 0.64 cm^−1^. Fourteen spectra recorded under similar conditions were averaged to yield a spectrum with a satisfactory ratio of signal to noise. For experiments with added NO/NO_2_, the resolution is 2.0 cm^−1^ (full width half maximum 2.6 cm^−1^) and one spectrum was recorded.

A small stream of O_2_ from a main stream at flow rate of *F*_O2_≅23 STP cm^3^ s^−1^ (STP denotes standard temperature 273 K and pressure 1 atm) served to purge the windows. A small stream of N_2_ (∼3.5 Torr in the reactor) was bubbled through liquid CH_3_CHI_2_ before entering the reactor. The total pressure was maintained in the range *P*_T_=80–85 Torr, with a partial pressure of *P*_CH3CHI2_≅0.28 Torr determined from its infrared absorption spectra. The average efficiency of photolysis of CH_3_CHI_2_ is estimated to be ∼8% according to an absorption cross-section of ∼3.1 × 10^−18^ cm^2^ per molecule (ref. 27) and a laser fluence of ∼7 × 10^16^ photons per cm^2^ at 308 nm. To obtain a desirable pressure of CH_3_CHI_2_, the samples and the flow reactor were heated to 328 K with heated water circulated from a thermostated bath through the jacket of the reactor. CH_3_CHI_2_ (96–98%), O_2_ (99.99%) and N_2_ (99.9995%) were used as received.

### Computational

The harmonic and anharmonic vibrational wavenumbers have been computed using quadratic force field obtained with the MP2 (ref. 34) and the NEVPT2 methods^35^ implemented in MOLPRO quantum chemistry package^36^, using a complete active space defined by four highest occupied orbitals and four lowest unoccupied orbitals; no symmetry has been used in these calculations to avoid problems during numerical determination of force-field coefficients. The anharmonic wavenumbers^37^ have only an approximate character, as the effects of three- and four-mode couplings have been neglected due to great computational complexity.

For CH_3_CHOO, we also employed the MULTIMODE method. See Supplementary Methods for more details. The MULTIMODE calculations employ full-dimensional potential energy surfaces (PESs) for *syn*-CH_3_CHOO and *anti*-CH_3_CHOO conformers. Most geometries are generated from classical dynamical trajectories with density functional theory (B3LYP). More points are chosen randomly around the equilibrium geometry and the torsion saddle point. To further increase the accuracy of the PESs, additional points are chosen along the grids of normal mode along the fully relaxed torsion path for both *syn*- and *anti*-CH_3_CHOO. Finally, we get about 39,000 *ab initio* energies, which are computed with the CCSD(T)-F12b method^38^,^39^ and aug-cc-pVDZ basis for carbon and oxygen atoms, and cc-pVDZ basis for hydrogen atoms.

The PESs are fitted with permutationally invariant method, which has been described in detail somewhere else^30^,^40^. The Morse variable is applied in the PES function, in which *y*_ij_=exp(–*r*_ij_/*α*) and *α* is fixed at 2 bohr. The maximum polynomial order is 5 for both PES and the number of coefficients is 5,801. The fitting root mean squares of the PESs are 0.076 and 0.068 cm^−1^ for *syn*- and *anti*-CH_3_CHOO, respectively. The comparisons of energies and harmonic frequencies between the PES and CCSD(T)-F12b calculation for *syn*- and *anti*-conformer are given in Supplementary Tables 9 and 10. As seen, great agreement is achieved between the PES and direct *ab initio* calculation. In addition, the dipole moment surfaces, which are computed with MP2 methods, are fitted as well for the infrared intensity calculation.

The infrared intensities are calculated using the dipole transition matrix and each element is determined by integrating the ground and vibrationally excited-state wave functions and the dipole moment components. The calculated energies and intensities of fundamental states for *syn*- and *anti*-CH_3_CHOO are listed in Supplementary Tables 1 and 2. Several fundamental states, especially the CH-stretching modes, are difficult to assign due to their strong coupling with other modes.

Rotation constants of the ground vibrational state and fundamental vibrational states are determined rigorously from calculated energy difference of *J*=1 and 0. The rotation constants of *syn*-CH_3_CHOO ground vibrational state agree with those determined experimentally^24^. The computed rotation constants for *syn*- and *anti*-CH_3_CHOO are shown in Supplementary Tables 7 and 8, respectively. These small energy differences are sensitive to the treatment of the torsion degree of freedom, and so several sets of calculations, including ones that eliminated that degree of freedom, were performed. The rotation constants that appear to be most consistent with the simulation of experimental bands are from the smaller set of calculation with 13 modes coupling for *syn*-CH_3_CHOO (14 modes for *anti*) and these are given in the tables.

## Author contributions

H.Y.L. and Y.H.H. performed the experiments and H.Y.L. analysed the data. X.W. performed the MULTIMODE calculations. Y.N. performed calculations with other methods. J.B. and H.A.W. conceived and designed the calculations, and contributed to writing sections of the paper. Y.P.L. conceived and designed the experiments and wrote a major part of the paper.

## Additional information

**How to cite this article:** Lin, H.-Y. *et al*. Infrared identification of the Criegee intermediates *syn*- and *anti*-CH_3_CHOO, and their distinct conformation-dependent reactivity. *Nat. Commun*. 6:7012 doi: 10.1038/ncomms8012 (2015).

## Supplementary Material

Supplementary Information Supplementary Figures 1-7, Supplementary Tables 1-10, Supplementary Methods and Supplementary References

## Figures and Tables

**Table 1:** Comparison of experimentally observed wavenumbers (cm^−1^) and intensities with the vibrational wavenumbers and infrared intensities of representative vibrational modes of CH_3_CHOO predicted with the MULTIMODE method.

*Symbol*	*Syn-CH*_*3*_*CHOO*			*Anti-CH*_*3*_*CHOO*			Description*
	*Mode*	*Experiment*	*Calculation*	*Mode*	*Experiment*	*Calculation*	
A'	*ν*_4_	1,476.8 (30)†	1,494 (8)†	*ν*_4_	1,479.0 (14)†	1,488 (10)†	CO str./HCO bend
A'	*ν*_7_	1,280.8 (40)	1,285 (19)	*ν*_7_	1,279.4 (17)	1,295 (3)	HCO bend/CO str.
A'	*ν*_8_	1,090.6 (10)	1,097 (6)	*ν*_8_		1,136 (1)	CH_2_ wag/CCH bend
A'	*ν*_9_	∼956.0 (−)	969 (5)	*ν*_10_	851.8 (73)	894 (49)	CCH bend/CH_2_ wag
A'	*ν*_10_	871.2 (100)	908 (100)	*ν*_9_	883.7 (100)	944 (100)	OO str.
A'	*ν*_12_		314 (3)‡	*ν*_12_		330 (7)	CCO/COO *iph* bend
A”	*ν*_17_		449 (0)	*ν*_17_		255 (0)§	*op* deformation
A”	*ν*_18_		208 (1)‡	*ν*_18_		156 (0)§	CH_3_ torsion

*Ip*, in-plane; *iph*, in-phase; *op*, out-of-plane; str., stretch.

^*^Approximate mode description. For *anti*-CH_3_CHOO, the HCO bending mode is replaced with CH *ip* bending mode for *ν*_7_, *ν*_8_ is mainly CCH bend, the CCH bend is replaced with OO stretch for *ν*_10_, and the *iph* bend is replaced with *oph* bend for *ν*_12_.

^†^Relative infrared intensities normalized to the most intense line. The maximal intensities of *syn*- and *anti*-CH_3_CHOO are predicted to be 44.4 and 43.6 km mol^−1^, respectively, with the MULTIMODE method (18-mode coupling); values of 13-mode coupling for *syn*- and 14-mode coupling for *anti*-CH_3_CHOO are 50.3 and 61.1 km mol^−1^, respectively.

^‡^Harmonic vibrational wavenumbers, as the anharmonic treatment of this mode is problematic using the methods employed here. Anharmonic vibrational wavenumbers are *ν*_12_=290 cm^−1^ and *ν*_18_=221 cm^−1^ predicted with the VCI/NEVPT2(8,8)/aug-cc-pVDZ method.

^§^Harmonic vibrational wavenumbers, as the anharmonic treatment of this mode is problematic using the methods employed here. Anharmonic vibrational wavenumbers are *ν*_17_=378 cm^−1^ and *ν*_18_=241 cm^−1^ predicted with the VCI/NEVPT2(8,8)/aug-cc-pVDZ method.
