# Supplementary material for: Infrared identification of the Criegee intermediates syn- and anti-CH3CHOO, and their distinct conformation-dependent reactivity
Source: Nat Commun. 2015 May 11;6:7012. doi: 10.1038/ncomms8012 (PMC4432623; doi:10.1038/ncomms8012)
Supplement: Supplementary Information — Supplementary Figures 1-7, Supplementary Tables 1-10, Supplementary Methods and Supplementary References [file ncomms8012-s1.pdf]

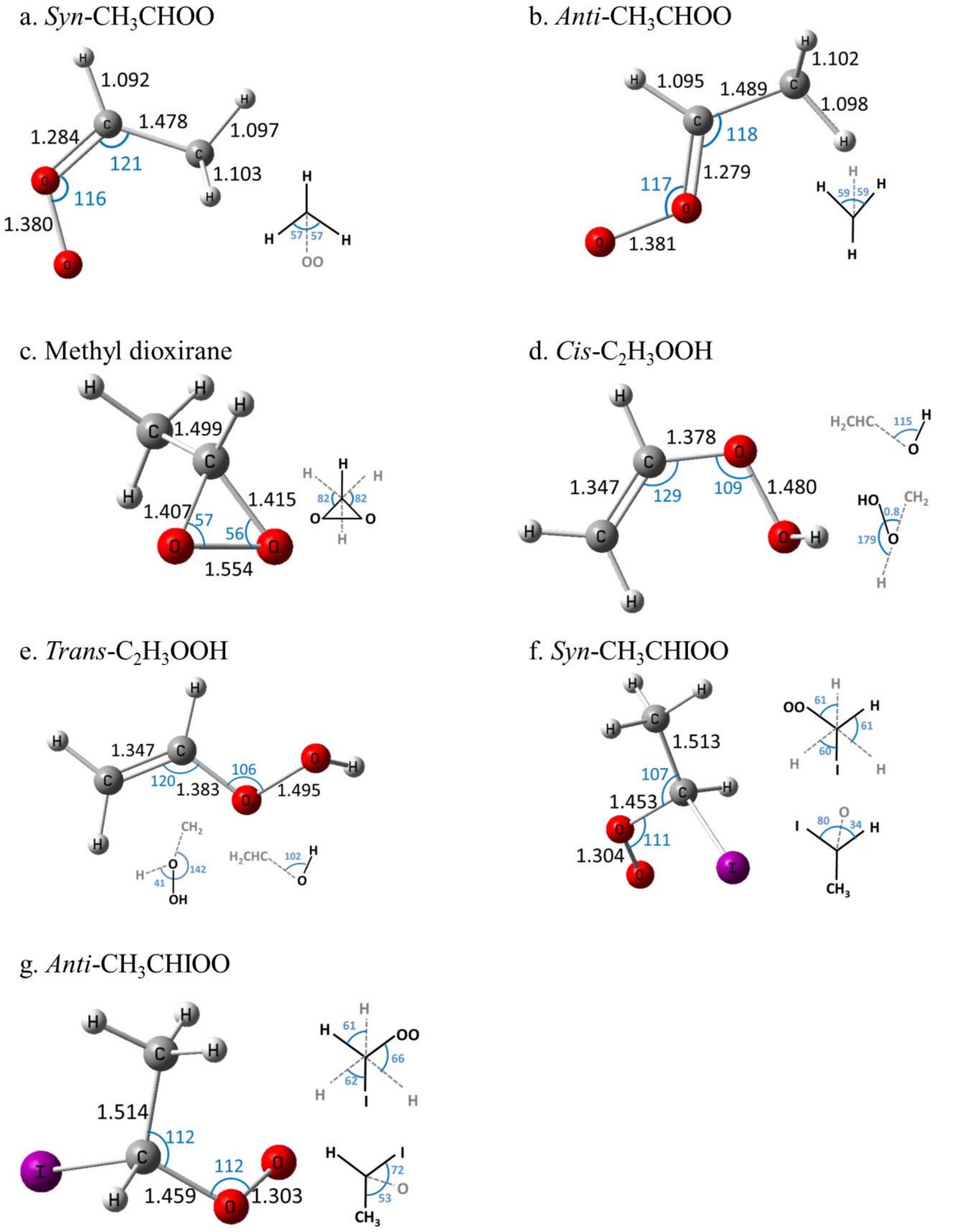

Supplementary Figure 1 | Geometries of possible carriers of the observed spectrum. a, *syn*-CH_3CHOO, **b,** *anti*-CH_3CHOO, **c,** methyl dioxirane, **d,** *cis*-vinyl hydroperoxide, and **e,** *trans*-vinyl hydroperoxide predicted with the NEVPT2(8,8)/aug-cc-pVDZ method, and **f,** *syn*-CH_3CHIOO and **g,** *anti*-CH_3CHIOO predicted with the NEVPT2(1,1)/aug-cc-pVDZ method. The bond distances are given in Å (black) and the bond angles are given in degree (blue).

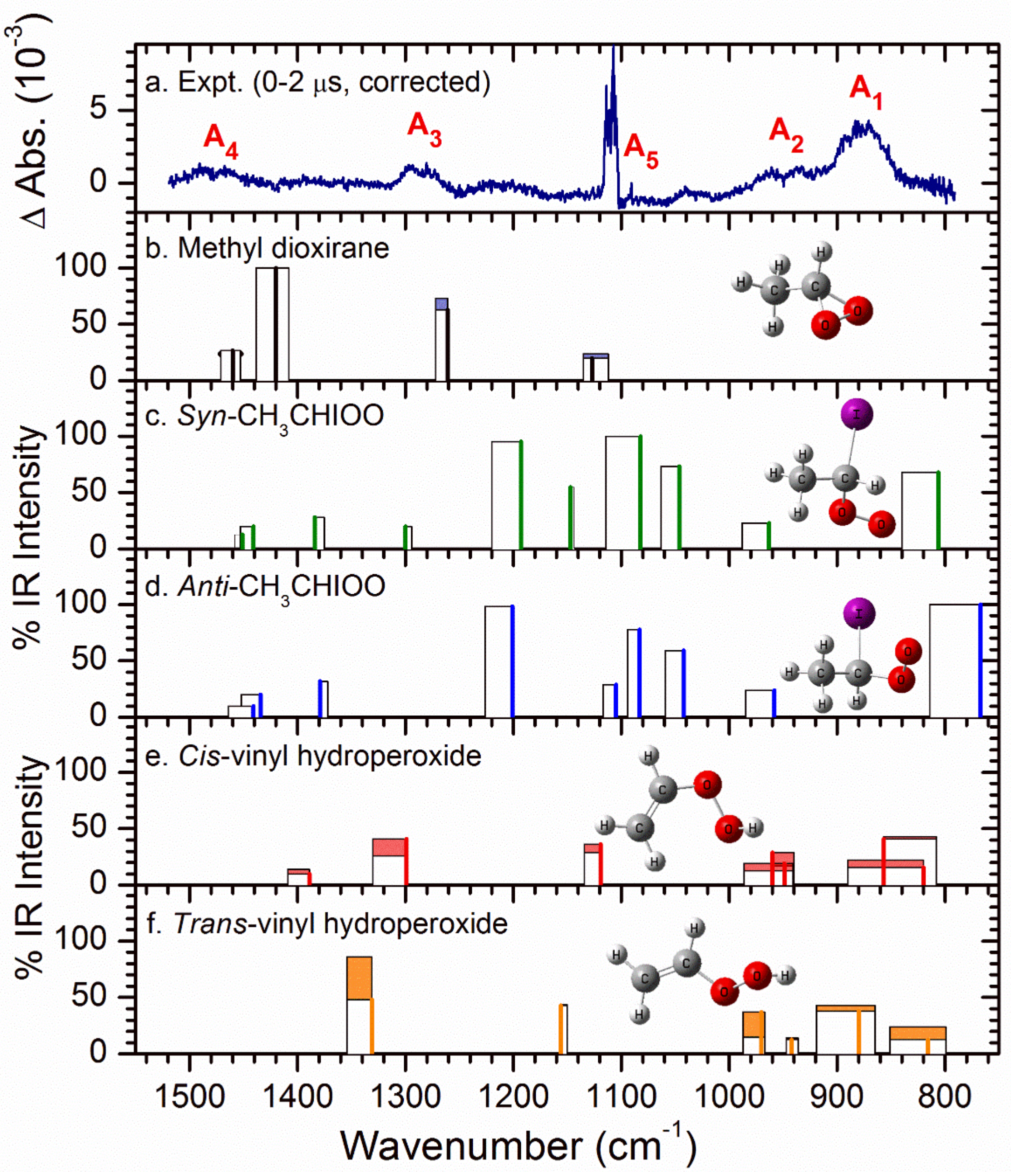

Supplementary Figure 2 | Comparison of observed spectra with predicted stick spectra of possible carriers in the $CH_3CHOO + O_2$ system. a, experimental spectrum taken from Fig. 1D. New features are indicated as A_1–A_5. Resolution is 0.5 cm^{-1}. **b,** possible ranges of anharmonic vibrational wavenumbers and IR intensities (Tables S3–S5) of methyl dioxirane, **c,** *syn*-CH_3CHIOO, **d,** *anti*-CH_3CHIOO, **e,** *cis*-vinyl hydroperoxide, and **f,** *trans*-vinyl hydroperoxide predicted with various methods shown as boxes. Thick lines indicate those predicted with the B3LYP/aug-cc-pVTZ-pp method (for CH_3CHIOO) or the VSCF/MP2/aug-cc-pVDZ method (for methyl dioxirane and vinyl hydroperoxide).

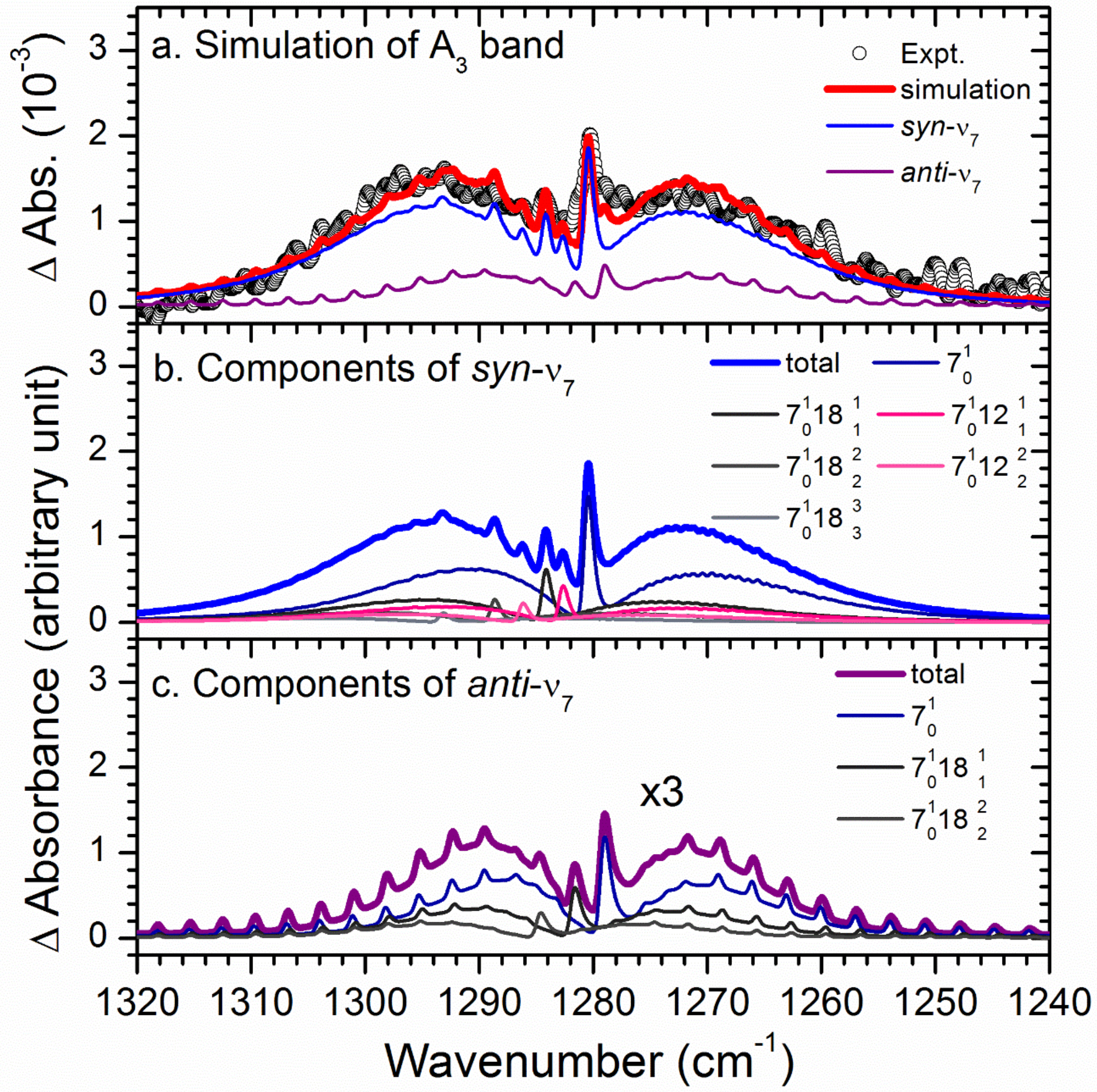

Supplementary Figure 3 | Spectral simulation of band A$_3$. a, comparison of experimental data (open circles) with simulated spectrum (thick solid red line); contributions of ν_7 of *syn*-CH_3CHOO and *anti*-CH_3CHOO are shown with thin lines. Spectral width is 0.64 cm^{-1}. **b,** contributions of fundamental and hot bands of ν_7 of *syn*-CH_3CHOO. **c,** contributions of fundamental and hot bands of ν_7 of *anti*-CH_3CHOO.

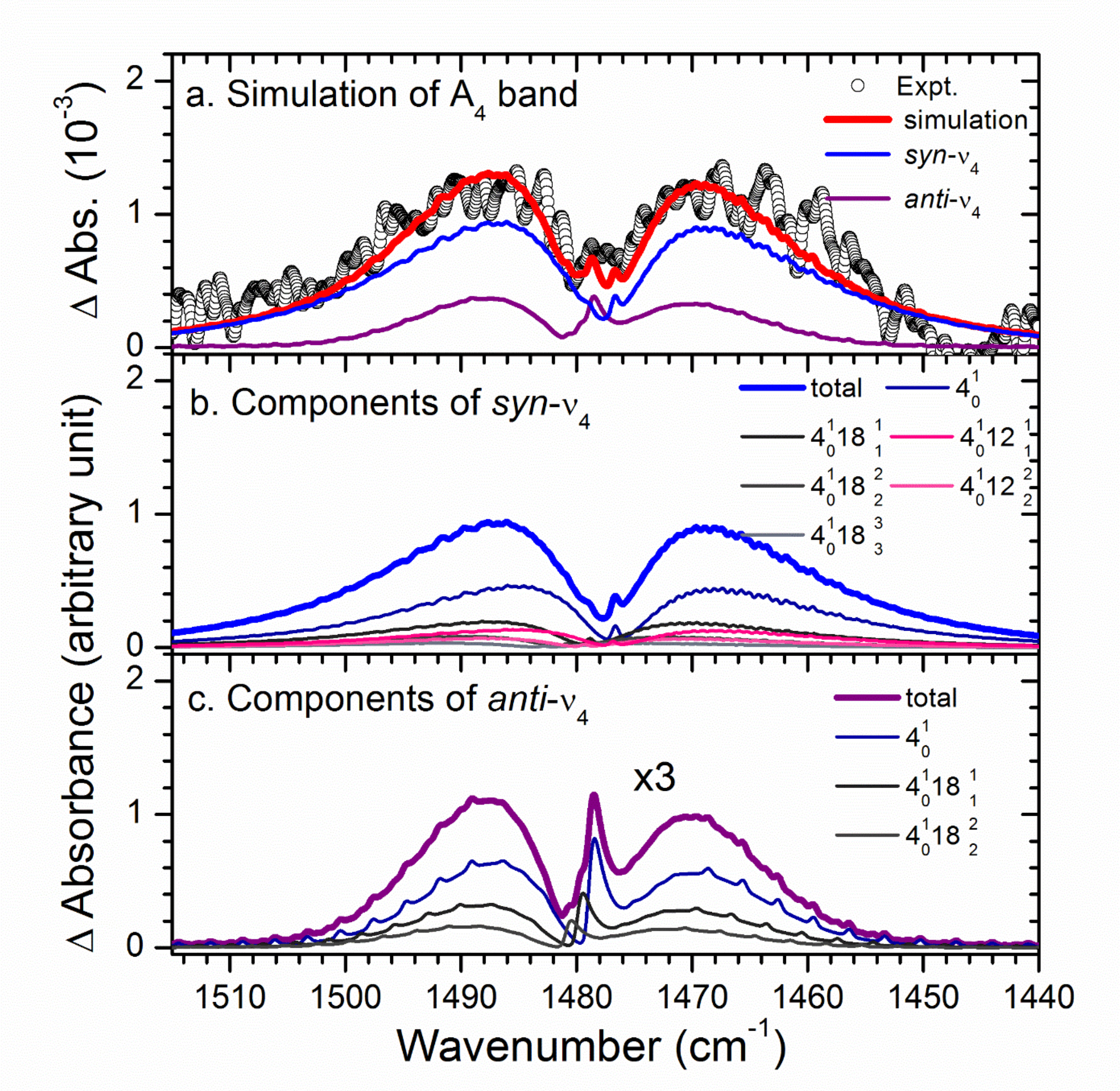

Supplementary Figure 4 | Spectral simulation of band A_4. a, comparison of experimental data (open circles) with simulated spectrum (thick solid red line); contributions of ν_4 of *syn*-CH_3CHOO and *anti*-CH_3CHOO are shown with thin lines. Spectral width is 0.64 cm^{-1}. **b,** contributions of fundamental and hot bands of ν_4 of *syn*-CH_3CHOO. **c,** contributions of fundamental and hot bands of ν_4 of *anti*-CH_3CHOO.

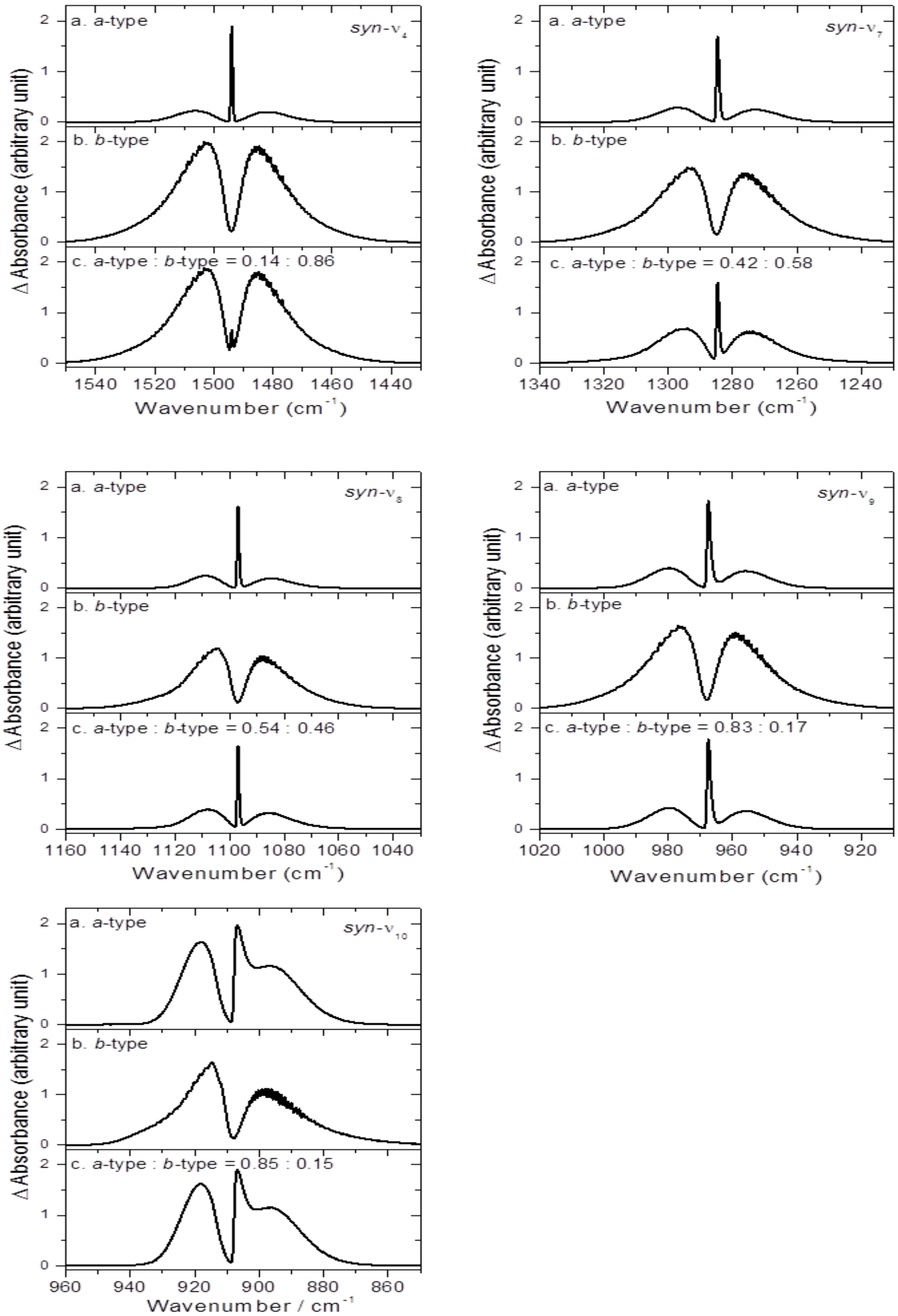

Supplementary Figure 5 | Simulation of *a*-, *b*-type, and the resultant spectra for the ν_4 and ν_7–ν_{10} modes of *syn*-CH_3CHOO. Parameters are: J_{max} = 170, T = 328 K, and Gaussian width = 0.64 cm^{-1}; type ratios are listed in figures.

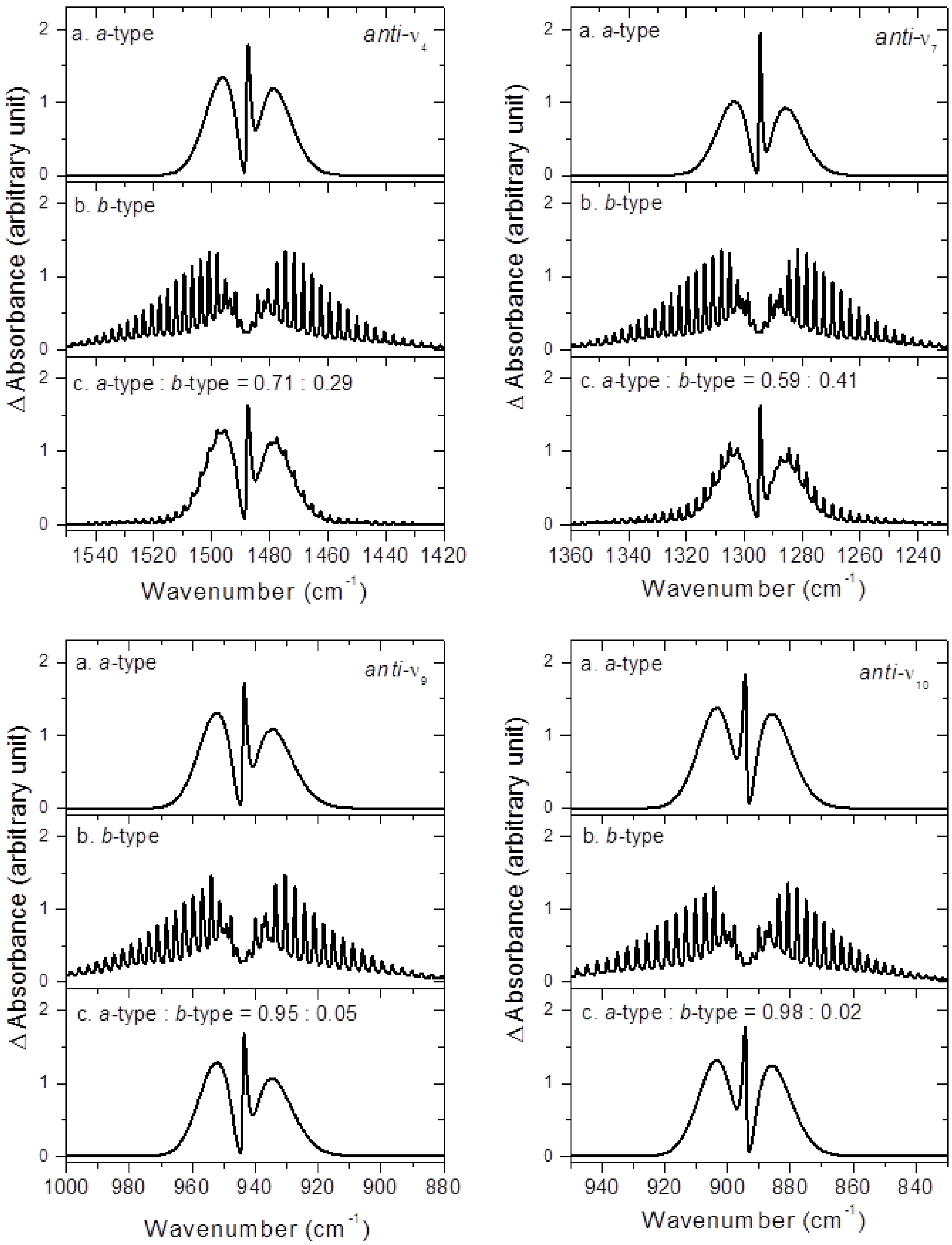

Supplementary Figure 6 | Simulation of *a*-, *b*-type, and the resultant spectra for the ν_4, ν_7, ν_9 and ν_{10} modes of *anti*-CH_3CHOO. Parameters are: J_{max} = 170, T = 328 K, and Gaussian width = 0.64 cm^{-1}; type ratios are listed in figures.

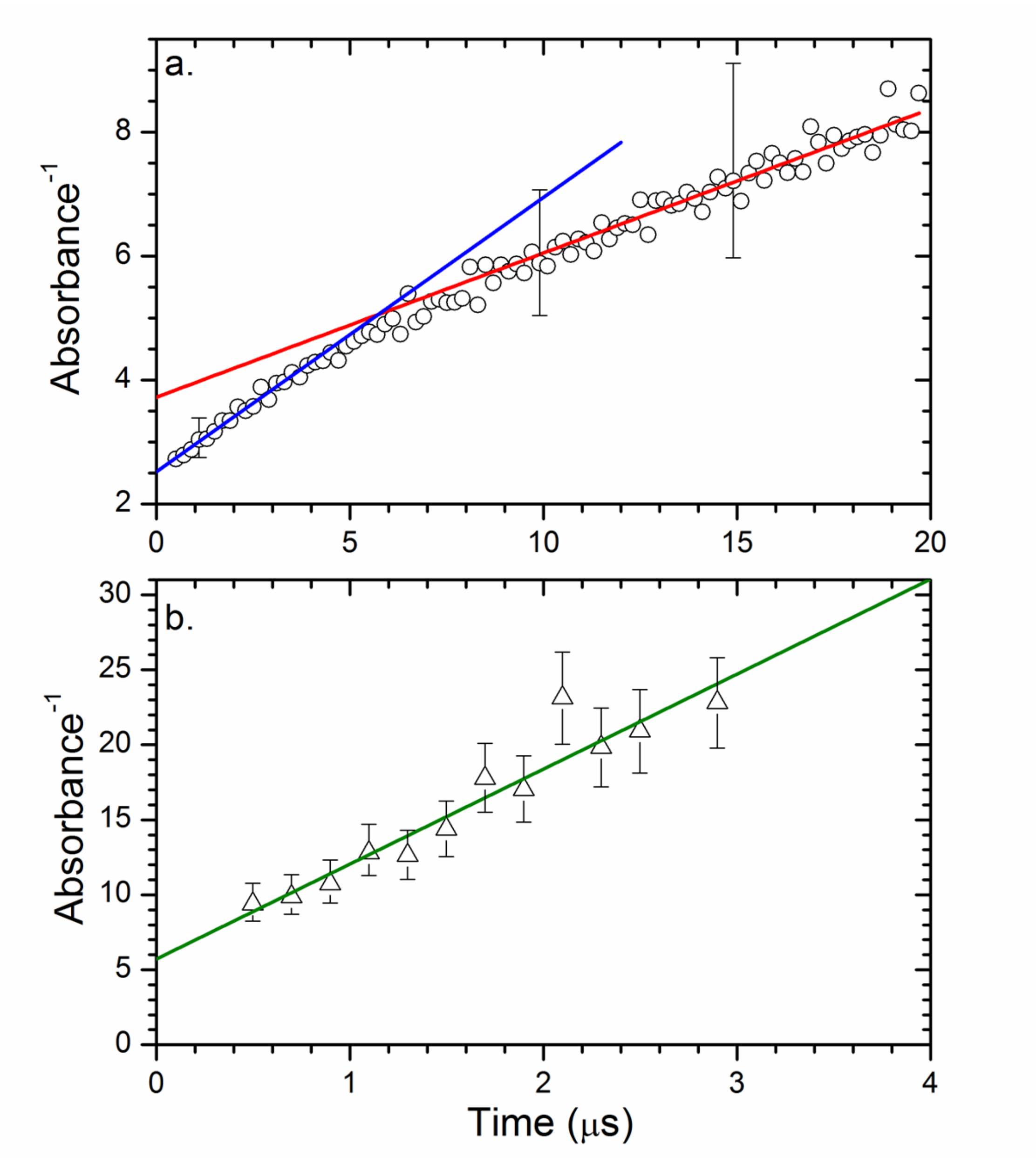

Supplementary Figure 7 | Plot of the reciprocal integrated absorbance versus reaction time. a, reciprocal integrated absorbance of band A_1 versus reaction time *t*. Data for $t \geq 12$ μs, assumed to be mainly due to *syn*-CH_3CHOO, are fitted with a line (red) with a slope 0.23±0.02 cm μs^{-1}, whereas data for $t \leq 4$ μs are fitted with a line (blue) with a slope 0.44±0.02 cm μs^{-1}. **b,** Reciprocal integrated absorbance of *anti*-CH_3CHOO versus reaction time *t* estimated by assuming that *syn*-CH_3CHOO dominates after 10 μs and that the fitted (red) line in Fig. 5a represents Γ^{-1} for *syn*-CH_3CHOO. Data are fitted with a line with a slope 6.3±0.6 cm μs^{-1}.

Supplementary Table 1 | Comparison of anharmonic vibrational wavenumbers (cm^{-1}) and IR intensities of vibrational modes of *syn*-CH_3CHOO predicted with various methods.

Mode	Sym.	MP2 /aug-cc-pVDZ	B3LYP /aug-cc-pVTZ	NEVPT2(8,8) /aug-cc-pVDZ	MULTIMODE (18 modes)	Mode description[a]
ν_1	A'	3114 (1)[b]	3098 (4)[b]	3119	3103 (1)[b]	CH str.
ν_2	A'	3035 (2)	2996 (11)	3030	3048 (0)	CH/CH_2 oph-str.
ν_3	A'	2843 (0)	2891 (5)	2853		CH_3 s-str.
ν_4	A'	1465 (6)	1538 (36)	1484	1494 (8)	CO str./HCO bend
ν_5	A'	1422 (1)	1414 (11)	1437	1431 (3)	CH_2 scissor.
ν_6	A'	1341 (2)	1361 (18)	1353	1371 (3)	CH_3 umbrella
ν_7	A'	1241 (27)	1319 (12)	1291	1285 (19)	HCO bend/CO str.
ν_8	A'	1106 (2)	1079 (25)	1112	1097 (6)	CH_2 wag/CCH bend.
ν_9	A'	980 (0)	962 (8)	972	969 (5)	CCH bend/ CH_2 wag
ν_{10}	A'	891 (100)	875 (100)	889	908 (100)	OO str.
ν_{11}	A'	695 (0)	665 (4)	668	676 (1)	CCO/COO oph-bend
ν_{12}	A'	308 (1)	291 (11)	290	314 (3)[c]	CCO/COO iph-bend
ν_{13}	A''	2834 (0)	2897 (1)	2871	2976 (0)	CH_2 a-str.
ν_{14}	A''	1405 (3)	1383 (14)	1427	1408 (4)	CH_2 twist.
ν_{15}	A''	1000 (0)	1019 (0)	1011	1008 (0)	op-deform
ν_{16}	A''	697 (4)	740 (22)	684	723 (7)	CH op-bend.
ν_{17}	A''	474 (0)	452 (0)	435	449 (0)	op-deform
ν_{18}	A''	214 (0)	109 (4)	221	208 (1)[c]	CH_3 torsion

[a]Approximate mode description. *a*: asymmetric; *s*: symmetric; str.: stretch; iph: in-phase; oph: out-of-phase; ip: in-plane; op: out-of-plane.
[b]Percentage IR intensities relative to the most intense line; the IR intensities are 460, 83, and 44 km mol^{-1} from MP2, B3LYP (harmonic) and MULTIMODE methods, respectively.
[c]Harmonic wavenumbers.

Supplementary Table 2 | Comparison of anharmonic vibrational wavenumbers (cm^{-1}) and IR intensities of vibrational modes of *anti*-CH_3CHOO predicted with various methods.

Mode	Sym.	MP2 /aug-cc-pVDZ	B3LYP /aug-cc-pVTZ	NEVPT2(8,8) /aug-cc-pVDZ	MULTIMODE (18 modes)	Mode description[a]
ν_1	A'	3052 (0)[b]	2990 (4)[b]	3097		CH/CH oph-str.
ν_2	A'	3009 (1)	2966 (1)	3021		CH/CH iph-str.
ν_3	A'	2899 (2)	2919 (2)	2917		CH_3 s-str.
ν_4	A'	1483 (23)	1521 (1)	1500	1488 (10)[b]	CO str./CH ip-bend.
ν_5	A'	1434 (1)	1425 (11)	1453	1427 (0)	CH_2 scissor.
ν_6	A'	1383 (5)	1395 (12)	1379	1390 (6)	CH_3 umbrella
ν_7	A'	1310 (1)	1319 (12)	1296	1295 (3)	CH ip-bend/CO str.
ν_8	A'	1143 (1)	1128 (3)	1155	1136 (1)	CCH bend
ν_9	A'	874 (100)	943 (100)	961	944 (100)	OO str.
ν_{10}	A'	926 (35)	872 (20)	892	894 (49)	OO str./CH_2 wag
ν_{11}	A'	575 (0)	547 (7)	560	565 (2)	CCO/COO iph-bend
ν_{12}	A'	323 (6)	322 (8)	340	330 (7)	CCO/COO oph-bend
ν_{13}	A"	2885 (0)	2914 (2)	2895		CH_2 a-str.
ν_{14}	A"	1441 (2)	1423 (9)	1456	1446 (3)	CH_3 deform
ν_{15}	A"	1043 (0)	1047 (0)	1043	1034 (0)	op- deform
ν_{16}	A"	845 (1)	850 (7)	822	840 (3)	CH op-bend
ν_{17}	A"	308 (0)	249 (0)	378	255 (0)[c]	op-deform
ν_{18}	A"	290 (0)	140 (1)	241	156 (0)[c]	CH_3 torsion

[a]Approximate mode description. *a*: asymmetric; *s*: symmetric; str.: stretch; iph: in-phase; oph: out-of-phase; ip: in-plane; op: out-of-plane.

[b]Percentage IR intensities relative to the most intense line; the IR intensities are 410, 121 and 44 km $mol^{-1}$from MP2, B3LYP (harmonic) and MULTIMODE methods, respectively.

[c]Harmonic wavenumbers

Supplementary Table 3 | Comparison of anharmonic vibrational wavenumbers (cm^{-1}) and IR intensities of vibrational modes of methyl dioxirane predicted with various methods.

Mode	B3LYP[a]	MP2[b]	NEVPT2(8,8)[b]
ν_1	2994 (17)[c]	3019 (16)[c]	3050
ν_2	2947 (46)	2967 (50)	2968
ν_3	2949 (8)	2954 (10)	2968
ν_4	1453 (24)	1460 (27)	1471
ν_5	1408 (100)	1420 (100)	1438
ν_6	1370 (6)	1361 (7)	1374
ν_7	1272 (73)	1261 (63)	1261
ν_8	1112 (24)	1127 (20)	1135
ν_9	923 (2)	921 (0.5)	920
ν_{10}	774 (3)	730 (2)	684
ν_{11}	457 (19)	463 (23)	468
ν_{12}	2956 (16)	2944 (15)	3061
ν_{13}	1445 (11)	1450 (10)	1460
ν_{14}	1182 (0.0)	1197 (3)	1198
ν_{15}	1009 (14)	1021 (23)	1026
ν_{16}	814 (22)	874 (27)	833
ν_{17}	383 (0.0)	394 (0.0)	397
ν_{18}	211 (0.3)	299 (0.4)	301

[a]With basis set aug-cc-pVTZ.

[b]With basis set aug-cc-pVDZ.

[c]Percentage IR intensities relative to the most intense line; the IR intensities are 63 and 55 km mol^{-1} from the B3LYP (harmonic) and MP2 methods, respectively.

Supplementary Table 4 | Comparison of relative energy, anharmonic vibrational wavenumbers (cm^{-1}) and IR intensities of vibrational modes of *trans*- and *cis*-vinyl hydroperoxide (C_2H_3OOH) predicted with various methods.

	trans-C_2H_3OOH			*cis*-C_2H_3OOH		
Mode	B3LYP[a]	MP2[b]	NEVPT2(8,8)[b]	B3LYP[a]	MP2[b]	NEVPT2(8,8)[b]
ν_1	3552 (57)[c]	3484 (34)[c]	3438	3564 (40)[d]	3461 (40)[d]	3395
ν_2	3115 (2)	3118 (1)	3122	3123 (1)	3131 (0.5)	3141
ν_3	3048 (3)	3057 (4)	3122	3040 (1)	3075 (1)	3080
ν_4	3065 (1)	3070 (1)	3076	3000 (0.2)	3077 (0.1)	3089
ν_5	1643 (75)	1631 (66)	1644	1641 (100)	1648 (98)	1662
ν_6	1392 (5)	1384 (3)	1391	1409 (14)	1389 (10)	1403
ν_7	1341 (86)	1331 (48)	1354	1330 (26)	1299 (41)	1298
ν_8	1299 (1)	1281 (2)	1288	1304 (2)	1288 (2)	1300
ν_9	1150 (44)	1156 (43)	1157	1134 (29)	1119 (36)	1133
ν_{10}	967 (15)	970 (37)	987	941 (13)	949 (19)	986
ν_{11}	947 (14)	942 (13)	936	940 (17)	960 (29)	951
ν_{12}	865 (43)	880 (38)	919	866 (22)	820 (16)	890
ν_{13}	851 (24)	816 (13)	799	833 (43)	857 (41)	808
ν_{14}	691 (6)	732 (6)	745	697 (4)	726 (4)	744
ν_{15}	522 (5)	514 (11)	504	608 (4)	611 (4)	610
ν_{16}	343 (4)	367 (2)	372	309 (0.4)	330 (1)	325
ν_{17}	211 (100)[e]	579 (100)	576	232 (4)	276 (5)	256
ν_{18}	85 (2)	143 (15)	140	156 (88)[e]	702 (100)	686
Energy / kJ mol^{-1}	3.18	2.96		0	0	

[a]With basis set aug-cc-pVTZ. [b]With basis set aug-cc-pVDZ. [c]Percentage IR intensities relative to the most intense line; the IR intensities are 118 and 105 km mol^{-1}from B3LYP (harmonic) and MP2 methods, respectively. [d]Percentage IR intensities relative to the most intense line; the IR intensities are 138 and 117 km mol^{-1}from B3LYP (harmonic) and MP2 methods, respectively. [e]Harmonic wavenumbers; VPT2 technique fails to produce anharmonic wavenumber for this mode.

Supplementary Table 5 | Comparison of relative energy, anharmonic vibrational wavenumbers (cm^{-1}) and IR intensities of vibrational modes of *syn*-CH_3CHIOO and *anti*-CH_3CHIOO predicted with various methods.

Mode	*syn*-CH_3CHIOO		*anti*-CH_3CHIOO	
	B3LYP[a]	NEVPT2(1,1)[b]	B3LYP[a]	NEVPT2(1,1)[b]
ν_1	3013 (3)[c]	3000	3015 (2)[c]	3005
ν_2	2994 (5)	3029	3002 (1)	2991
ν_3	2971 (10)	2972	2979 (12)	3016
ν_4	2939 (23)	3162	2934 (24)	2941
ν_5	1451 (13)	1458	1441 (10)	1464
ν_6	1441 (20)	1453	1434 (20)	1452
ν_7	1384 (28)	1375	1379 (32)	1372
ν_8	1300 (20)	1294	1334 (5)	1329
ν_9	1193 (95)	1220	1201 (98)	1226
ν_{10}	1147 (55)	1144	1105 (29)	1117
ν_{11}	1082 (100)	1114	1083 (78)	1094
ν_{12}	1046 (73)	1063	1042 (59)	1059
ν_{13}	963 (23)	988	958 (24)	985
ν_{14}	806 (68)	840	767 (100)	814
ν_{15}	550 (83)	578	626 (5)	648
ν_{16}	478 (50)	517	493 (98)	533
ν_{17}	349 (3)	364	321 (12)	340
ν_{18}	249 (5)	278	255 (2)	319
ν_{19}	235 (3)	294	236 (2)	316
ν_{20}	216 (1)	634	222 (0.2)	152
ν_{21}	65 (3)	95	75 (1)	111
Energy / kJ mol^{-1}	0	0	3.14	0.40

[a]With basis set aug-cc-pVTZ-pp

[b]With basis set aug-cc-pVDZ.

[c] Percentage IR intensities (harmonic) relative to the most intense line; the IR intensities of *syn*- and *anti*-CH_3CHIOO are 40 and 41 km mol^{-1}, respectively.

Supplementary Table 6 | Comparison of fitted wavenumbers (cm^{-1}) and relative intensities of various vibrational modes of *syn*-CH_3CHOO and *anti*-CH_3CHOO with MULTIMODE predictions.

Transition	Intensity	Energy	Expt.	Caln 1[a]	Caln2[b].	Transition	Intensity	Energy	Expt.	Caln1[a, c]
Band A_1						Band A_3				
syn-CH_3CHOO						*syn*-CH_3CHOO				
10^1_0	1.00		871.2	911.9	908.1	7^1_0	1.00		1280.8	1287.0
$10^1_0 18^1_1$	0.43	193	+2.4	+5.0	+13.6	$7^1_0 18^1_1$	0.42	198	+3.7	+7.8
$10^1_0 18^2_2$	0.25	316	+6.3	+12.0	+29.4	$7^1_0 18^2_2$	0.18	391	+8.2	+17.2
$10^1_0 18^3_3$	0.10	525	+11.8			$7^1_0 18^3_3$	0.07	597	+12.7	
$10^1_0 12^1_1$	0.29	282	+10.1	+12.0	+28.3	$7^1_0 12^1_1$	0.29	282	+2.2	
$10^1_0 12^2_2$	0.15	433	+15.8	+11.1	+80.8	$7^1_0 12^2_2$	0.15	433	+5.7	
anti-CH_3CHOO						*anti*-CH_3CHOO				
9^1_0	1.00		883.7	954.6	944.2	7^1_0	1.00		1279.4	1299.8
$9^1_0 18^1_1$	0.52	149	+1.8	+18.1	+21.7	$7^1_0 18^1_1$	0.50	158	+2.6	+17.2
$9^1_0 18^2_2$	0.29	282	+5.3	+27.7	+45.1	$7^1_0 18^2_2$	0.25	316	+5.6	+22.0
$9^1_0 17^1_1$	0.35	239	+2.3	+8.6	+20.4	Band A_4				
$9^1_0 17^2_2$	0.15	433	+6.3			*syn*-CH_3CHOO				
anti-CH_3CHOO						4^1_0	1.00		1476.8	1495.9
10^1_0	1.00		851.8	902.7	894.2	$4^1_0 18^1_1$	0.42	198	+2.2	+6.8
$10^1_0 18^1_1$	0.50	158	+9.2	+16.9	+22.8	$4^1_0 18^2_2$	0.18	391	+4.2	+12.6
$10^1_0 18^2_2$	0.25	316	+15.2	+27.6	+47.1	$4^1_0 18^3_3$	0.07	597	+6.2	
$10^1_0 17^1_1$	0.35	239	+11.2	+8.7	+25.3	$4^1_0 12^1_1$	0.29	282	+0.2	
$10^1_0 17^2_2$	0.15	433	+20.2		+61.7	$4^1_0 12^2_2$	0.15	433	+2.7	
						anti-CH_3CHOO				
						4^1_0	1.00		1479.0	1498.2
						$4^1_0 18^1_1$	0.50	158	+1.0	+14.1
						$4^1_0 18^2_2$	0.25	316	+2.0	

[a]MULTIMODE calculations coupling 15 modes of *syn*-CH_3CHOO or 16 modes of *anti*-CH_3CHOO, which includes 13 modes of *syn* (14 modes for anti) as discussed in the text and another two lowest frequency modes. The maximal excitations of two lowest energy modes are limited to 2.

[b]Calculations coupling all 18 modes of *syn*-CH_3CHOO or *anti*-CH_3CHOO.

[c]Hot band results are problematic, see text for discussion.

Supplementary Table 7 | Comparison of rotational parameters of *syn*-CH_3CHOO in their ground and vibrationally excited states predicted with various methods.

	MP2			B3LYP			MULTIMODE[a]		
	A'/*A*"	*B*'/*B*"	*C*'/*C*"	*A*'/*A*"	*B*'/*B*"	*C*'/*C*"	*A*'/*A*"	*B*'/*B*"	*C*'/*C*"
ν_4	0.9989	0.9984	0.9985	0.9966	0.9986	0.9978	0.9973<br>(0.9998)[b]	0.9981<br>(0.9985)	0.9980<br>(0.9992)
ν_7	0.9979	1.0023	1.0010	1.0001	1.0012	1.0008	0.9982<br>(0.9985)	0.9981<br>(0.9983)	0.9980<br>(0.9989)
ν_8	1.0008	1.0001	0.9986	1.0065	0.9994	0.9986	1.0011<br>(1.0003)	1.0006<br>(0.9977)	0.9980<br>(0.9946)
ν_9	0.9993	0.9961	0.9975	0.9994	0.9968	0.9975	0.9966<br>(0.9967)	0.9992<br>(0.9990)	0.9989<br>(0.9992)
ν_{10}	0.9922	1.0002	0.9967	0.9914	1.0005	0.9967	0.9904<br>(0.9856)	0.9990<br>(0.9985)	0.9946<br>(0.9946)
	A"	*B*"	*C*"	*A*"	*B*"	*C*"	*A*"	*B*"	*C*"
$\nu = 0$	0.6015	0.2393	0.1764	0.5876	0.2333	0.1719	0.5810	0.2388	0.1748
Expt.[c]	0.5866	0.2379	0.1744	0.5866	0.2379	0.1744	0.5866	0.2379	0.1744

[a] MULTIMODE calculation coupling 13 modes of *syn*-CH_3CHOO, see text for details.

[b]Ratios used in the simulation for the best fit are listed in parentheses.

[c]From reference*24*.

Supplementary Table 8 | Comparison of rotational parameters of *anti*-CH_3CHOO in their ground and vibrationally excited states predicted with various methods.

	MP2			B3LYP			MULTIMODE[a]		
	A'/A''	B'/B''	C'/C''	A'/A''	B'/B''	C'/C''	A'/A''	B'/B''	C'/C''
ν_4	1.0000	0.9993	0.9991	0.9837	0.9991	0.9977	0.9966	1.0000	0.9993
							(0.9954)[b]	(1.0000)	(0.9986)
ν_7	1.0003	1.0005	1.0000	1.0021	1.0002	0.9997	0.9978	1.0000	1.0000
							(0.9974)	(1.0000)	(0.9993)
ν_8	1.0067	0.9990	0.9979	0.9987	0.9992	0.9977	1.0051	0.9997	0.9982
							(1.0050)	(0.9993)	(0.9978)
ν_9	1.0039	0.9996	0.9987	0.9891	0.9979	0.9963	0.9950	0.9990	0.9982
							(0.9959)	(0.9987)	(0.9978)
ν_{10}	1.0038	0.9958	0.9941	1.0087	0.9987	0.9965	1.0049	0.9997	0.9982
							(1.0051)	(0.9993)	(0.9986)
	A''	B''	C''	A''	B''	C''	A''	B''	C''
$\nu = 0$	1.6336	0.1491	0.1402	1.6272	0.1472	0.1380	1.5998	0.1482	0.1392
Expt.[c]	1.6176	0.1479	0.1390	1.6176	0.1479	0.1390	1.6176	0.1479	0.1390

[a] MULTIMODE calculation coupling 13 modes of *syn*-CH_3CHOO, see text for details.

[b]Ratios used in the simulation for the best fit are listed in parentheses.

[c]From reference *24*.

Supplementary Table 9 | The comparison of energies (cm^{-1}) and harmonic vibrational wavenumbers (cm^{-1}) of *syn*-CH_3CHOO between the fitted PES and direct *ab initio* calculations. SP refers to the torsion saddle point.

	Minimum		SP	
	ab initio	PES	*ab initio*	PES
Energies	0.00	0.00	730.3	730.4
Mode				
ν_1	3204.2	3206.0	3194.8	3196.8
ν_2	3161.2	3161.2	3177.2	3176.7
ν_3	3029.5	3030.5	3040.7	3036.7
ν_4	1527.4	1528.3	1539.7	1538.4
ν_5	1466.5	1468.0	1487.7	1484.6
ν_6	1397.7	1396.0	1388.8	1389.2
ν_7	1316.6	1317.9	1328.9	1329.2
ν_8	1115.4	1115.8	1157.8	1156.4
ν_9	977.6	977.5	929.8	933.2
ν_{10}	922.1	922.7	883.0	875.4
ν_{11}	677.6	677.8	653.3	648.0
ν_{12}	308.0	307.5	335.9	332.7
ν_{13}	3084.7	3085.3	3103.7	3105.8
ν_{14}	1449.2	1449.9	1449.8	1446.0
ν_{15}	1031.3	1034.0	1037.7	1046.2
ν_{16}	720.6	721.0	757.7	752.4
ν_{17}	447.7	448.3	430.8	435.1
ν_{18}	201.8	206.0	175.9i	177.1i

Supplementary Table 10 | The comparison of energies (relative to *syn*-CH_3CHOO) and harmonic vibrational wavenumbers (cm^{-1}) of *anti*-CH_3CHOO between PES and *ab initio* calculation. SP refers to the torsion saddle point.

	Minimum		SP	
	ab initio	PES	*ab initio*	PES
Energies	1274.2	1274.2	1700.9	1700.9
Mode				
ν_1	3168.3	3168.6	3171.2	3167.6
ν_2	3154.0	3153.6	3152.4	3156.1
ν_3	3039.2	3039.3	3047.0	3056.1
ν_4	1533.8	1534.6	1507.2	1515.8
ν_5	1466.0	1466.5	1483.8	1485.5
ν_6	1419.8	1419.8	1414.7	1408.7
ν_7	1321.3	1321.5	1318.1	1312.4
ν_8	1159.2	1159.4	1147.9	1142.3
ν_9	956.0	957.0	1002.1	1003.0
ν_{10}	904.6	904.9	890.6	890.9
ν_{11}	560.4	560.4	558.9	556.8
ν_{12}	324.5	324.3	325.1	326.1
ν_{13}	3103.6	3103.4	3115.6	3111.6
ν_{14}	1476.8	1477.1	1470.2	1469.0
ν_{15}	1053.7	1054.2	1053.2	1064.7
ν_{16}	850.2	848.3	822.7	819.8
ν_{17}	251.0	251.3	242.1	242.6
ν_{18}	156.2	155.7	171.1i	171.3i

Supplementary Methods

Spectral simulation. With the PGopher program,[1] we simulated the spectrum of each band using experimental rotational parameters *A*", *B*", and *C*" reported by Nakajima and Endo,[2] ratios of rotational parameters *A*'/*A*", *B*'/*B*", and *C*'/*C*" and *a*-type/*b*-type ratios of each mode predicted with the MULTIMODE method, as listed in Table S7 and S8, J_{max} = 170, *T* = 328 K, and a Gaussian width 0.64 cm^{-1}. For an improved fitting, the ratios of *A*'/*A*", *B*'/*B*", and *C*'/*C*" were changed slightly from the theoretical values, as shown in parentheses in Supplementary Tables 7 and 8. Simulated *a*- and *b*-type spectra for ν_4 and ν_7–ν_{10} of *syn*- CH_3CHOO and ν_4, ν_7, ν_9, and ν_{10} of *anti*-CH_3CHOO are shown in Supplementary Figs 5 and 6. The projections of the dipole derivatives for each vibrational mode of CH_3CHOO onto rotational axes *a*, *b* and *c* determine the weighting of bands of types *a*, *b*, and *c* in each vibrational absorption band. The position of each band was varied to achieve the best fit.

Additional computational details. MULTIMODE is a program for vibrational calculation, which has been discussed in detailed and applied to various systems.[3] Generally speaking, the MULTIMODE calculation uses Watson Hamiltonian with mass-scaled normal coordinates. The key feature of MUTLMODE is to expand the full potential to a hierarchy *n*-mode representation, which greatly reduces the multi-dimensional integration. The CH_3CHOO molecule has 18 degrees of freedom, and we use 4-mode representation in our study.

The Watson Hamiltonian is rigorous for semi-rigid molecules, which is not obviously the case for *syn*- and *anti*-CH_3CHOO with the internal torsion motion. However, from a recent Diffusion Monte Carlo calculation,[4] the ground state wave functions are localized, and can be applied with the semi-rigid approach. Since the energies of highly excited states of torsion mode exceed the torsion barrier height, 730 and 427 cm^{-1} for *syn*- and *anti*- CH_3CHOO, respectively, the excitation

of torsion mode is restricted to 2 to avoid any unreasonable couplings with torsion mode. The semi-rigid treatment for *anti*-CH_3CHOO is more problematic, especially for "hot bands", than for *syn*-CH_3CHOO because the barrier to internal rotation for the *anti*-conformer is roughly 300 cm^{-1} lower than for the *syn*-conformer.[4]

Thirteen harmonic oscillator basis are used for each mode in the first vibrational self-consistent field (VSCF) calculation. Then the VSCF virtual states are used as the basis-function for the following vibrational configuration-interaction (VCI) step. The size of VCI calculation is restricted by the maximum excitation of each mode and maximum sum of excitations of all modes. In the current study, all 18 vibration modes of CH_3CHOO are included, and up to 4 modes can be excited simultaneously. As mentioned above, the maximum excitation of torsion mode is 2. To reduce the expense of calculation, the total excitation of four CH-stretching modes of CH_3CHOO is limited to 3. Since the frequencies of CH-stretching modes are far beyond the spectral range investigated, 800–1500 cm^{-1}, such restrictions on CH stretch modes affect little the accuracy of the calculation. The maximum excitations of all other modes are 7. Cs symmetry is applied in the MULTIMODE calculation. The sizes of VCI matrix for the two Cs blocks are 22,157 and 17,924.

In addition, we performed another smaller set of calculations which only includes 13 modes of *syn*- and 14 modes of *anti*-conformer to compare with the calculations that includes all 18 modes. For *syn*-conformer, the frequencies of nine vibration modes are in the measured spectral range from 800 to 1500 cm^{-1}, and ten such modes for *anti*-conformer. The smaller set calculation of 13 modes for *syn*-CH_3CHOO includes such nine modes and four CH stretch modes, and similarly for calculation of the 14 modes for *anti*-CH_3CHOO.

The hot-band transition can be calculated from MULTIMODE calculation as well. We present two sets of calculation results in Supplementary Table 6. One is from the calculation with all 18 modes coupling; another is from the smaller set of calculation with selected 13 modes for *syn*-conformer (14 modes for *anti*) as well as another two lowest frequency modes. As seen, relatively large deviation of the hot-band transition shift is observed between the experiment and MULTIMODE prediction. One main reason is the treatment of the torsional mode of CH_3CHOO. As mentioned above, the maximum excitation of torsion mode is restricted to 2. Such restriction affects the calculated energies of torsion states and other modes with small energies. In addition, including the low-frequency modes enhances the coupling among states. For the ground state, the torsion splitting is small and such restriction has negligible effect to calculation. However, as the torsion mode is excited to the fundamental or overtone states, our "single-reference" treatment of torsion motion may be problematic. Nevertheless, the blue-shifted hot-band transition of these bands agrees with experiment simulation.

Supplementary References

1. Western, C. M. PGOPHER, a program for simulating rotational structure, University of Bristol UK, version 7.1.108 (2010), http://pgopher.chm.bris.ac.uk.

2. Nakajima, M. & Endo, Y. Communication: Spectroscopic characterization of an alkyl substituted Criegee intermediate *syn*-CH_3CHOO through pure rotational transitions. *J. Chem. Phys.* **140,** 011101 (2014).

3. Bowman, J. M., Carter, S. & Huang, X. MULTIMODE: A code to calculate rovibrational energies of polyatomic molecules. *Int. Rev. Phys. Chem.* **22,** 533–549 (2003).

4. Bowman, J. M., Wang, X. & Homayoon, Z. Ab initio Computational Spectroscopy and Vibrational Dynamics of Polyatomic Molecules: Applications to *syn* and *anti*-CH_3CHOO and NO_3. *J. Molec. Spectros* (2015). http://dx.doi.org/10.1016/j.jms.2014.12.012
